# Cognition, Depression, Pain, and Exercise Motives as Predictors of Longitudinal Profiles of Physical Activity During a Seven‐Year Follow‐Up Among Older Adults

**DOI:** 10.1111/sms.14777

**Published:** 2024-12-13

**Authors:** Saila Kyrönlahti, Jenni Lehtisalo, Tiia Ngandu, Miia Kivipelto, Timo Strandberg, Riitta Antikainen, Tiina Laatikainen, Hilkka Soininen, Jaakko Tuomilehto, Satu Havulinna, Jenni Kulmala

**Affiliations:** ^1^ Department of Public Health, Lifestyles and Living Environments Unit Finnish Institute for Health and Welfare (THL) Helsinki Finland; ^2^ Faculty of Social Sciences (Health Sciences) and Gerontology Research Center (GEREC) Tampere University Tampere Finland; ^3^ Division of Clinical Geriatrics, Department of Neurobiology, Care Sciences and Society, Center for Alzheimer Research Karolinska Institute Stockholm Sweden; ^4^ Faculty of Health Sciences, Institute of Public Health and Clinical Nutrition University of Eastern Finland Kuopio Finland; ^5^ Ageing Epidemiology Research Unit School of Public Health Imperial College London London UK; ^6^ Center for Life Course Health Research/Geriatrics University of Oulu Oulu Finland; ^7^ University of Helsinki and Helsinki University Hospital Helsinki Finland; ^8^ Medical Research Center Oulu Oulu University Hospital Oulu Finland; ^9^ Institute of Clinical Medicine – Neurology University of Eastern Finland Kuopio Finland; ^10^ Department of Public Health University of Helsinki Helsinki Finland; ^11^ South Ostrobothnia Central Hospital Seinäjoki Finland; ^12^ Department of Healthcare and Social Welfare, Services Unit Finnish Institute for Health and Welfare Helsinki Finland

**Keywords:** determinants of physical activity, geriatric intervention, longitudinal study, physical activity profiles

## Abstract

This study investigated longitudinal physical activity (PA) profiles over 7 years in the Finnish Geriatric Intervention Study to Prevent Cognitive Impairment and Disability (FINGER). Cognition, depression, pain, and PA motives were included as determinants of the PA profiles. The 1259 participants, aged 60–77 years at baseline, were randomized into either a control group receiving general health advice, or an intervention group offered a comprehensive 2‐year multidomain intervention including physical exercise, diet advice, cognitive training, and vascular risk factor management. The participants reported weekly moderate‐intensity PA at baseline and 1, 2, 5, and 7 years after the baseline. Those providing PA data at two or more time points were included (*n* = 1188). Longitudinal PA profiles were determined using latent class growth analysis, and their associations with baseline determinants via multinomial logistic regression analysis. Interaction terms were added to investigate whether the intervention modified these associations. Six PA profiles were identified: *Very high–stable* (6%), *High–stable* (22%), *Moderate–declining* (47%), *Moderate–steeply declining* (5%), *Low–increasing* (9%), and *Constantly low* (12%). Participants in the intervention group and those motivated by distal and proximal benefits of exercise were likelier to maintain high PA level. Conversely, depressive symptoms and pain were predictors of *Constantly low profile*. Results show that high baseline PA was generally maintained, while greater variability in PA changes was observed among initially less active participants.

## 1 Background

Physical activity (PA) is crucial for promoting healthy aging, enhancing both physical and cognitive functioning, whereas inactivity increases the risk of adverse health events [1]. Although PA is considered one of the most important factors affecting the aging process [2], maintaining consistent activity levels becomes challenging with age. Over a quarter of the global adult population fails to meet recommended PA levels [3], with the risk of inactivity substantially higher after age 65 [4].

Research shows that PA patterns among aging adults vary; while some maintain a consistent level of activity, others experience declines or completely cease regular PA [5, 6, 7]. Changes such as retirement may encourage an increase in PA in some older adults [8], while factors like declining physical and mental health and pain are known correlates of low PA. However, less is known about how cognition, depressive symptoms, and pain affect PA in the long term, or about the motives that encourage older adults to engage in and sustain PA as they age.

Pain is common in older adults [9] and often leads to reduced PA, as activities may be avoided from fear of exacerbating pain [10, 11]. The model of fear‐avoidance behavior [11] describes a possible mechanism by which pain may in long‐term lead to inactivity: the perceived threat of worsening pain may lead to avoidance of movement and activity, which in turn can lead to a sharp decrease in functional capacity and further hinder PA. Nonetheless, PA may mitigate both the occurrence and sensitivity to pain [10, 12].

The associations between cognition and depressive symptoms with PA may also be bidirectional. Convincing evidence has linked PA to reduced dementia risk at late life, with active older adults more likely to preserve cognitive functions [13, 14, 15]. Significant mental health benefits have also been shown from being physically active [16, 17]. Because cognitive functions and depressive symptoms affect various aspects of behavior [18, 19] and decision‐making [20, 21], they may be important for initiating and maintaining PA in the long term. However, the findings on whether, and to what extent, cognitive impairment and depressive symptoms hinder long‐term engagement in PA among older adults are unclear. Some studies have found that they inversely affect PA levels [22, 23, 24, 25, 26], while others suggest that the evidence on reverse causation is less clear [27, 28, 29]. Furthermore, prior evidence is mostly based on observational studies. Whether providing an intensive PA and lifestyle intervention can improve PA also among older people experiencing pain, depressive symptoms, or cognitive decline is an important study question.

Knowledge of the health benefits of PA alone is insufficient to motivate increased activity [30]. Therefore, studying what motivates engagement in PA is also critical. While maintaining or improving health and functional ability are important motives for engaging in PA among older adults [31, 32, 33], the social aspects related to PA, such as social interaction or encouragement from others, a sense of community, and having friends and family who exercise, are also important facilitators [32, 33, 34]. Reasons to engage in PA can differ significantly with age, with older individuals more often reporting reasons related to social interactions and enjoyment as main motives, while younger adults cite specific goals like fitness or stress reduction [33]. Less is known about how different reasons for PA relate to each other and predict PA engagement in long‐term among older adults and whether a multidomain lifestyle intervention modifies these associations.

The Finnish Geriatric Intervention Study to Prevent Cognitive Impairment and Disability (FINGER) showed that a multi‐domain intervention, including guided physical exercise, diet counseling, cognitive training, and vascular risk factor management, could enhance cognitive and physical performance in adults at risk of dementia [35, 36, 37]. While research has shown that interventions can increase PA during active intervention phases, their role in fostering long‐term PA engagement remains unclear. In designing targeted and effective intervention programs to promote sustained PA, it is important to understand the longitudinal patterns of PA as well as the barriers and reasons for PA, and whether the intervention can modify these factors.

The current study aims to investigate the variations in PA engagement over a 7‐year follow‐up among participants of the FINGER trial. Second aim is to investigate the role of the intervention, baseline cognition, depressive symptoms, pain, and PA motives as determinants of longitudinal PA profiles.

## 2 Methods

### 2.1 Study Population and Design

**FIGURE 1:**
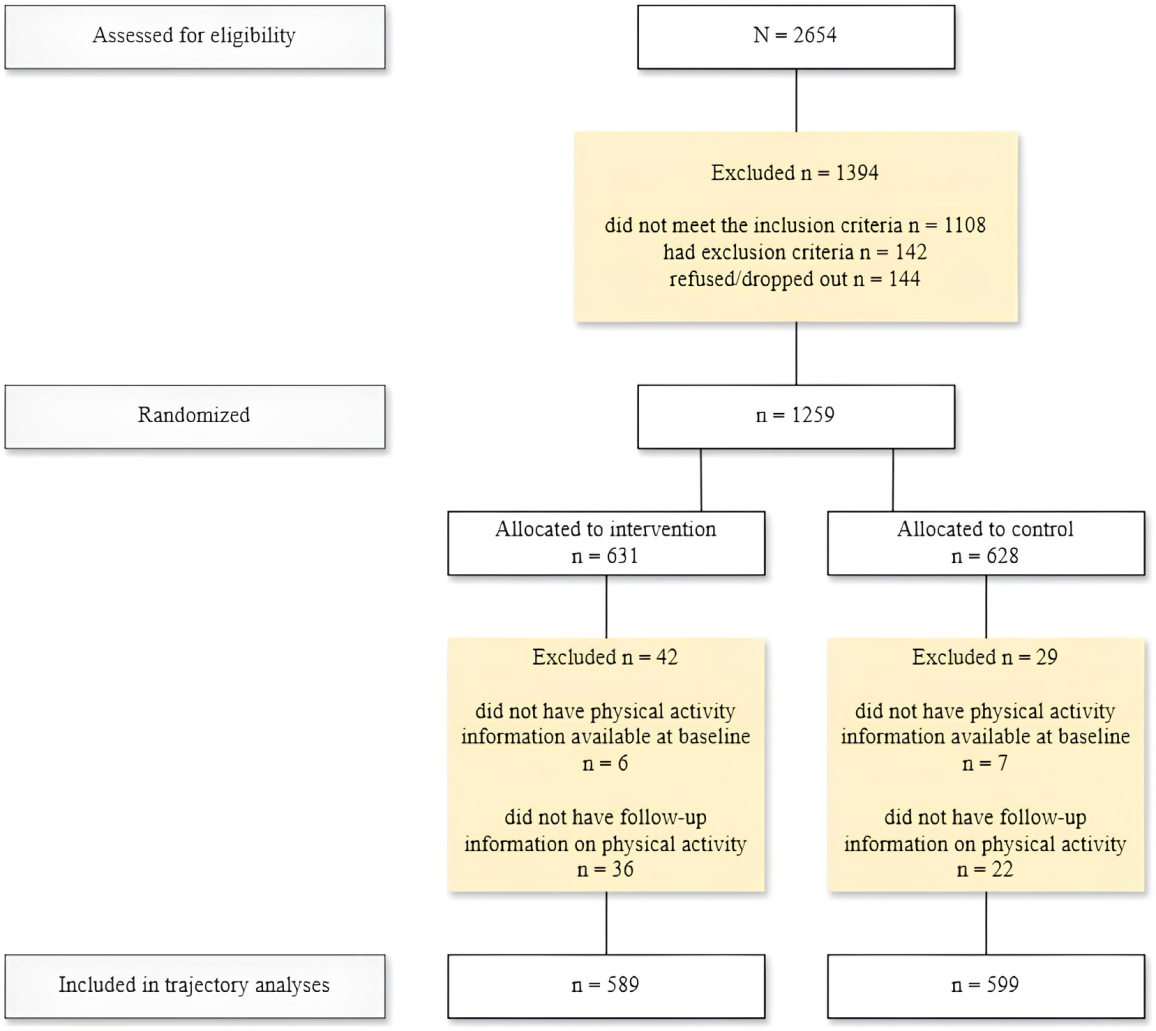
Flowchart of study population.

The FINGER is a multidomain randomized controlled trial conducted in six cities in Finland. Participants were recruited from a cohort of people who had previously participated in population‐based, observational surveys [38, 39]. The study flowchart is presented in Figure 1. Altogether 2654 people were screened for eligibility. To be eligible, the person had to be 60–77 years old, have dementia risk score of six or more points as assessed by CAIDE (Cardiovascular Risk Factors, Aging and Dementia) risk score [40], and not meet any of the following exclusion criteria: previously diagnosed or suspected dementia, Mini Mental State Examination score < 20 points (out of the maximum 30 points), disorders that could prevent safe participation in the intervention, and simultaneous participation in another trial. Physical inactivity was not a prerequisite for study eligibility. However, the CAIDE risk score allocates an additional point to individuals who are inactive. Consequently, inactive participants may have been more likely to be included in the study. Eligible participants (*n* = 1260) were randomized into either intervention or control group (1:1). The participants were not actively informed about the randomization group they were allocated to [37].

Both groups received regular health advice. In addition, the intervention group received intervention that lasted for 2 years and included five components. The details of the multidomain intervention have been reported previously [35, 37]. In short, the intervention comprised physical exercise training program, nutritional guidance, cognitive training, social activities, and monitoring and management of cardiometabolic risk factors.

The physical exercise component in the intervention group was guided by the study physiotherapist at the gym. The training program, which was based on international guidelines, and modified from the Dose Responses to Exercise Training (DR's EXTRA) study protocol [41], was individually tailored, and it comprised progressive muscle strength training 1–3 times/week, independent aerobic exercise 2–5 times/week, and postural balance exercises. Strength training included eight exercises that targeted the body's main muscle groups (knee extension/flexion, abdomen and back muscle exercises, upper back and arm muscle exercises, rotation, and bench press). Progression of training was determined by repetition maximum measurements (conducted at baseline and at 1, 3, 6, 9, 12, 18, and 24 month). Aerobic training included individually preferred activities, with aerobic group activities also provided.

The analytic sample for the current study comprised FINGER participants who had baseline and at least one follow‐up observation on PA during the 7‐year follow‐up. The study sample for regression analyses was further restricted to the participants who had information available on all study variables used in analyses.

All the participants signed an informed consent. The study conforms to the 1975 declaration of Helsinki (as revised in 2008). The Coordinating Ethics Committee of the Hospital District of Helsinki and Uusimaa approved the study (approval number 94/13/03/00/2009). The FINGER trial's ClinicalTrials.gov Identifier is NCT01041989.

### 2.2 Assessment of PA

The frequency of engaging in PA was determined similarly at five time points (baseline, 1, 2, 5, and 7 years after baseline). Participants were asked how often they engaged in PA lasting 20 min or more and resulting in at least mild shortness of breath and sweating. The scale ranged from 1 (5 or more times/week) to 7 (injury or illness that does not enable to exercise). For the present analysis, answer options 7 and 6 (less than once/week) were combined and the scale was reversed; the final variable ranged from 0 to 5, higher numbers indicating higher engagement.

Respondents in the intervention group provided data on average at 4.28 (SD 0.98) timepoints of the possible five study waves, and the control group at 4.33 (SD 0.95) timepoints, respectively.

### 2.3 Independent Variables

The independent variables were measured at baseline. Demographic factors included age, sex (male/female), and education (years).

The cognitive assessment at baseline was carried out by a study psychologist with an extended version of the neuropsychological test battery (NTB) [42]. A composite score from 14 tests was standardized to the sample mean and SD.

Depressive symptoms were evaluated using the Zung Self‐rating Depression Scale [43]—a 20‐item, validated measure for the evaluation of the affective, psychological, and somatic symptoms related to depression [44]. Respondents were asked to rate their agreement with the statements on a Likert scale from 1 to 4, from which a total raw score ranging from 20 to 80 points was calculated. A previously defined cutoff point of 40 points was used to indicate absence or presence of depressive symptoms [45].

Pain was assessed with RAND‐36 bodily pain scale [46], which comprises two questions about pain severity and pain that interferes with one's normal work activities during the previous 4 weeks. RAND‐36 is a widely used instrument for assessing health‐related quality of life, with good psychometric properties [47, 48]. Both pain items were scored on a 0–100 range and averaged together [49]. The resulting composite score was standardized, and the scale was reversed so that higher *z*‐score indicates more severe and disturbing pain.

To examine the PA motives, the participants were asked a question: “Which of the following factors encourage you to exercise?” Respondents were instructed to select all those that applied to them from among twelve answer options (1 = positive health effects, 2 = encouragement from healthcare professionals, 3 = disease management, 4 = improvement of well‐being and mood, 5 = family and loved ones exercise, 6 = positive effects on appearance, 7 = information about exercising obtained from the media, 8 = meeting friends, 9 = exercising is trendy, 10 = exercising is fun, 11 = other reason, specify, and 12 = nothing). For the analyses, option 11 was excluded. We used a data‐driven approach to examine which motives are most important and how they relate to each other. Principal component analysis (PCA) was used to reduce the number of items into fewer components and to discover the underlying patterns of the reasons for PA. The factor scores for the extracted components were used as continuous variables in the explanatory analyses. The three derived components were (1) *Distal and proximal benefits of exercise*, (2) *Appearance and media influence*, and (3) *Disease management* (Table S1). The description of the PCA is provided in the Appendix S1.

### 2.4 Statistical Analysis

General estimation equation modeling was used to investigate the overall trajectories of PA during the follow‐up in the intervention and the control group and to calculate the mean estimates of PA frequency with their 95% CIs at each timepoint. The metric of time was years. Time and randomization groups with their interaction term were included into the model. To control for the intraindividual correlation between repeated measurement, an independent correlation structure was specified, in which the dependence between observations is included in the model through the observed correlation matrix [50].

Next, latent class growth analysis (LCGA) was conducted to characterize latent subgroups of participants with a similar developmental trajectory of PA during the follow‐up. Following Nagin's two‐step procedure [51], an increasing number of quadratic shaped trajectory models were fitted until the fit statistics showed no improvement in the model fit (Table S2). The optimal solution was determined based on statistical model fitting criteria [52], including Bayesian information criterion (BIC), Akaike information criterion (AIC), entropy, and the average posterior probabilities for class membership. The size and interpretability of the profiles were also considered. Next, whether the fit of the chosen model improved with cubic, linear, or intercept‐only shaped trajectories was tested [51].

The posterior probabilities of membership in each profile were obtained for each participant, who were then assigned in the profile to which they had the highest probability to belong to. The derived profiles were then related to the explanatory variables. The baseline characteristics for the total sample and by profiles were reported as means and standard deviations (SD) for continuous variables, and as proportions for categorical variables.

Next, multinomial logistic regression modeling was used to investigate if the allocation group (intervention vs. control), baseline cognition, depressive symptoms, pain, and motives were associated with the PA‐profiles. The models were adjusted for sociodemographic variables. Effect modification of the investigated associations by the allocation group was examined by adding the interaction term (independent variable * allocation group) to each model. It was assumed that data were missing at random, and all participants who were randomized and had at least one post‐baseline observation of PA, were included and analyzed according to the group they were originally assigned.

The results are presented as average marginal effects (AMEs) with their 95% CIs. AME describes the average difference in the probability of membership in each profile depending on the value of the independent variable. Stata Version 18.0 (StataCorp, College Station, TX, USA) was used to run the analysis.

## 3 Results

The demographic characteristics at baseline for the whole sample and by PA profiles are presented in Table 1. The mean age of the participants was 68.8 (SD 4.7) years. There were slightly less women than men in the sample (46.2%). The mean of years of education was 10.0 (SD 3.4) years.

**TABLE 1:** Baseline characteristics of participants by physical activity profiles in The Finnish geriatric intervention study to prevent cognitive impairment and disability (FINGER).

	All	Constantly low	Low—increasing	Moderate—declining	Moderate—sharply declining	High—stable	Very high—stable
N	1188	137	104	564	56	257	70
Age, years (SD)	68.8 (4.7)	69.1 (4.4)	69.2 (4.8)	68.7 (4.7)	70.5 (4.9)	68.8 (4.6)	67.9 (5.0)
Women, *n* (%)	549 (46.2)	51 (38.1)	59 (45.7)	292 (49.7)	28 (50.0)	98 (46.0)	21 (30.9)
Education, years (SD)	10.0 (3.4)	10.0 (3.5)	9.6 (3.1)	10.1 (3.4)	10.6 (4.4)	9.7 (3.3)	10.3 (3.7)
Intervention group, *n* (%)	589 (49.6)	64 (46.7)	39 (37.5)	283 (50.2)	23 (41.1)	144 (56.0)	36 (51.4)
Cognition (NTB *z*‐score; SD)	0.00 (0.57)	0.03 (0.57)	−0.06 (0.59)	0.02 (0.58)	0.00 (0.54)	−0.01 (0.57)	−0.09 (0.58)
Depressive symptoms (Zung total score; SD)	33.93 (7.51)	36.79 (8.30)	34.45 (7.30)	33.71 (7.19)	35.08 (8.23)	32.84 (7.22)	32.64 (7.81)
Pain (*z*‐score; SD)	0.00 (1.00)	0.37 (1.26)	0.04 (1.03)	−0.05 (0.94)	0.18 (0.98)	−0.16 (0.89)	−0.03 (1.0)
Motives							
Distal and proximal benefits of exercise (*z*‐score; SD)	0.56 (0.27)	0.27 (0.37)	0.44 (0.27)	0.59 (0.23)	0.49 (0.26)	0.66 (0.19)	0.67 (0.22)
Appearance and media influence (*z*‐score; SD)	0.26 (0.30)	0.16 (0.23)	0.17 (0.23)	0.27 (0.30)	0.24 (0.33)	0.32 (0.31)	0.26 (0.28)
Disease management (*z*‐score; SD)	0.13 (0.38)	0.20 (0.34)	0.17 (0.36)	0.11 (0.38)	0.26 (0.39)	0.08 (0.38)	0.12 (0.41)

*Note:* Baseline characteristics are calculated among participants with non‐missing data. Missing data included education (*n* = 1), cognition (*n* = 1), depressive symptoms (*n* = 124), and pain (*n* = 10).

Attrition analyses (Table S3) showed that those who dropped out did not differ significantly from the included group, except that their scores for *Distal and proximal benefits of exercise* were slightly lower.

### 3.1 Trajectories of PA

**FIGURE 2:**
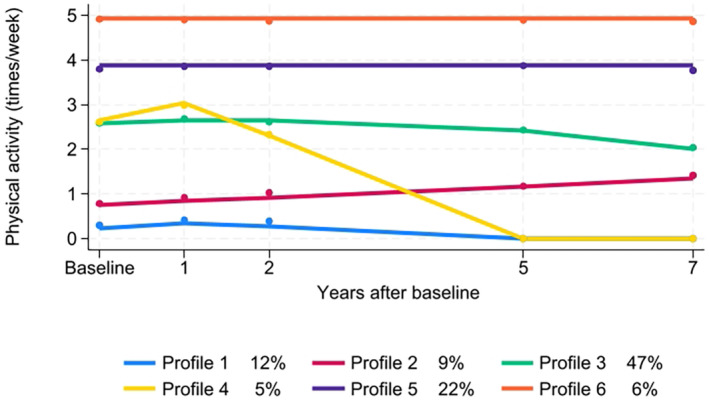
Latent profiles of physical activity among the participants of the Finnish Geriatric Intervention Study to Prevent Cognitive Impairment and Disability (FINGER) during a 7‐year follow‐up (*n* = 1188).

Six distinct PA‐profiles were identified among the whole sample (Figure 2). The best‐fitting solution comprised of three second‐order polynomial trajectories, one with a linear slope and two with intercept‐only trajectories (Table S1). Overall, the average posterior probabilities of group membership were relatively high (0.72–0.88), and the entropy value above the 0.70 threshold indicated a good class separation [52].

The most common profile was named *Moderate—declining* (Profile 3; 47%), where the mean frequency of PA at baseline was 2.6 times/week (SD 1.2). It remained stable at the moderate level during the first 5 years of follow‐up and after that PA declined to 2.0 times/week (SD 1.4) at the seven‐year follow‐up.

The second‐largest profile was *High—stable* (Profile 5; 22%), in which PA remained constant at a high baseline level (3.9 times/week, SD 1.2) across the follow‐up. Another smaller profile of the same shape (intercept‐only) was identified, *Very high—stable* (Profile 6; 6%), where the PA of the participants remained at a very high level throughout the 7‐year follow‐up period.

Approximately one out of ten participants were classified into *Constantly low* profile (Profile 1; 12%), in which PA at baseline was very low (0.3 times/week, SD 0.6), and despite a slight initial increase in the first year (0.5 times/week, SD 0.7), it remained at a very low level throughout the follow‐up. In *Low—increasing* (Profile 2; 9%), PA gradually increased during the follow‐up from initial baseline level 0.7 times/week (SD 0.8) to 1.5 times/week, (SD 1.4) at the end of the follow‐up. The smallest profile was *Moderate—sharply declining* (Profile 4; 5%), in which participants had moderate level PA at baseline (2.7 times/week, SD 1.6), which slightly increased during the first year to 3.2 times/week (SD 1.3), and then steeply decreased to an inactive level.

The overall trajectories of PA (times/week) among the intervention and the control group are depicted in Figure S1. At baseline, PA was similar among the intervention and the control group. During the first year, there was an increase in PA in the intervention group, but not in the control group. After one‐year follow‐up, PA declined in both groups, but it remained at a slightly higher level among the intervention group throughout the follow‐up. The difference in PA frequency was statistically significant at one‐year follow‐up (2.9 times/week (95% CI 2.7–3.0) vs. 2.5 times/week (95% CI 2.4–2.6) in the intervention and control group, respectively).

### 3.2 Predictors of PA‐Profiles

For regression analyses, *High—stable* and *Very high—stable* (with identical development of PA; intercept only) were combined into *High—stable profile*. Table 2 describes the AMEs for each independent variable.

**TABLE 2:** Associations of sociodemographic and baseline factors with longitudinal physical activity profiles among the participants of the Finnish geriatric intervention study to prevent cognitive impairment and disability (FINGER): Adjusted AME and their 95% confidence intervals (CI) from adjusted multinomial logistic regression models.

Independent variable	Constantly low	Low—increasing	Moderate—declining	Moderate—sharply declining	High—stable	*p* for interaction^a^
	AME (95% CI)	AME (95% CI)	AME (95% CI)	AME (95% CI)	AME (95% CI)	
Age^b^	0.00	0.00	0.00	**0.01 (0.00–0.01)**	0.00	0.728
Sex^b^						
Female^b^	ref	ref	ref	ref	ref	0.129
Male	**0.04 (0.00–0.08)**	0.00 (−0.04 to 0.03)	**−0.07 (−0.13 to −0.02)**	0.00 (−0.03 to 0.02)	0.04 (−0.01 to 0.09)	
Education^b^	0.00 (−0.01 to 0.00)	0.00 (−0.01 to 0.00)	0.00 (0.00–0.01)	0.00	(−0.01 to 0.01)	0.878
Group allocation^b^						
Control	ref	ref	ref	ref	ref	
Intervention	−0.01 (−0.05 to 0.02)	**−0.04 (−0.07 to −0.01)**	0.01 (−0.05 to 0.07)	−0.02 (−0.04 to 0.01)	**0.06 (0.01–0.11)**	
Cognition^c^	0.02 (−0.02 to 0.06)	−0.01 (−0.05 to 0.02)	0.00 (−0.06 to 0.06)	0.00 (−0.26 to 0.02)	0.00 (−0.06 to 0.05)	0.327
Depressive symptoms^c^						
Low (Zung score < 40)	ref	ref	ref	ref	ref	0.917
High (Zung score ≥ 40)	**0.09 (0.03–0.15)**	0.03 (−0.02 to 0.07)	−0.06 (−0.14 to 0.02)	0.02 (−0.02 to 0.05)	**−0.08 (−0.14 to −0.01)**	
Pain^c^	**0.04 (0.02–0.06)**	0.00 (−0.01 to 0.02)	−0.02 (−0.05 to 0.01)	0.01 (−0.01 to 0.02)	**−0.03 (−0.06 to −0.01)**	0.059
Motives^c^						
Distal and proximal benefits of exercise	**−0.33 (−0.38 to −0.27)**	**−0.15 (−0.20 to −0.10)**	0.08 (−0.03 to 0.20)	**−0.06 (−0.10 to −0.03)**	**0.46 (0.35–0.57)**	0.642
Appearance and media influence	**−0.15 (−0.23 to −0.07)**	**−0.10 (−0.17 to −0.03)**	0.08 (−0.02 to 0.18)	0.00 (−0.04 to 0.04)	**0.17 (0.09–0.25)**	0.840
Disease management	**0.05 (0.01–0.10)**	0.02 (−0.02 to 0.06)	−0.04 (−0.12 to 0.03)	**0.05 (0.02–0.08)**	**−0.08 (−0.15 to −0.02)**	**0.015**
Intervention group^d^	0.04 (−0.02 to 0.10)	−0.01 (−0.07 to 0.04)	0.10 (−0.01 to 0.21)	0.04 (−0.01 to 0.08)	**−0.17 (−0.27 to −0.07)**	
Control group^d^	**0.07 (0.01–0.14)**	**0.06 (0.00–0.12)**	**−0.20 (−0.31 to −0.10)**	**0.07 (0.02–0.11)**	0.00 (−0.09 to 0.09)	

*Note: N* = 1188. Omitted from regression models due to missing data: education *n* = 1, cognition *n* = 1, depressive symptoms *n* = 124, and pain *n* = 10. Estimates in bold are statistically significant at the *p* = 0.05 level.

^a^ Interaction term: allocation status * independent variable.

^b^ Adjusted for research center.

^c^ Additionally adjusted for age, sex, education, and allocation status.

^d^ Stratified analyses due to significant interaction term.

Age slightly increased the likelihood of membership in the *Moderate—sharply declining profile* but was not associated with the other profiles. Men were 7% less likely members in the *Moderate—declining profile*, and 4% more likely members in the *Constantly low profile* as compared with women. Education was not associated with the profiles. Being allocated to the intervention group decreased the likelihood of belonging to the *Low—increasing profile* by 4% and increased the likelihood of membership in the *Constantly high profile* by 6%.

After adjustments for the sociodemographic factors and the allocation status, baseline cognition was not associated with membership in any of the PA profiles. The likelihood of membership in *Constantly low profile* was 9% higher for those who had depressive symptoms as compared with those who did not. Moreover, one SD increase on pain z‐score increased the likelihood of membership in *Constantly low profile* by 4%.

Having depressive symptoms decreased the likelihood of belonging to *High—stable profile* by 8% and having one SD higher pain *z*‐score by 3%.

Overall, the motivation components were more strongly associated with the PA profiles than cognition, depression, or pain. One SD increase on *Distal and proximal benefits of exercise* score was associated with 46% increase in the likelihood of belonging to *High—stable profile*, and 33%, 15% and 6% decrease in the likelihood of membership in *Constantly low, Low—increasing*, and *Moderate—sharply declining profiles*, respectively. Also, less strong associations were found for *Appearance and media influence*: one SD increase on the score increased the likelihood of belonging to *High—stable profile* by 17% and decreased the likelihood of belonging to *Constantly low* and *Low increasing profiles* by 15% and 10%, respectively.

A statistically significant interaction term (*p* = 0.015) between *Disease management* and the allocation status, was found. Stratified analysis by the allocation status showed that in the control group one SD increase in the factor score increased the likelihood of belonging to the profiles with less optimal trajectories of PA by 6%–7%, whereas it decreased the likelihood of belonging to *Moderate—slightly declining profile* by 20%. In the intervention group, one SD increase in the factor score decreased the likelihood of belonging to the *High—stable profile* by 17%. Associations with the other profiles were statistically not significant.

## 4 Discussion

This study is one the few that have longitudinally mapped PA development in an elderly population at‐risk of dementia over an extended period. The study identified six longitudinal PA profiles, each with distinct developmental trajectories. The results highlight significant variability in how older adults maintain or change PA levels over time. While PA generally decreased over time, with an initial increase among the intervention group (Figure S1), heterogeneous trajectories of PA emerged (Figure 2). The derived profiles demonstrate that while highly active individuals at baseline maintained their activity levels, those initially less active exhibited varying trajectories. Nonetheless, most remained at least moderately active throughout the follow‐up with their trajectories demonstrating only minor decrease in activity level at the end of the follow‐up.

Although it is well known that PA decreases with age [3], longitudinal studies on PA patterns among older adults remain limited. Laddu et al. (2017) investigated PA trajectories over 7 years in men aged 65 and older, identifying three declining trajectories [6]. Our findings similarly show that baseline PA levels were key in differentiating groups, with most participants following a moderate‐declining trajectory. However, we observed greater heterogeneity, including profiles with increasing and sharply decreasing trajectories not seen in their study [6]. Another study [7] on women aged 70–79 years at baseline identified four PA trajectories over twelve years, including highly active individuals who maintained their activity and a group with moderate activity that slightly decreased. Consistent with our results, these trajectories were present, but our study found fewer participants in the constantly inactive and sharply declining groups. The observed differences likely stem from different study designs—the previous studies were observational [6, 7], capturing natural PA patterns, whereas our study used data from a multidomain lifestyle intervention trial which likely produced more variation in PA trajectories in our study compared to the previous studies. Notably, our study encompassed both men and women, possibly also contributing to the observed variability compared with prior studies focusing solely on one sex [6, 7].

Although the intervention group maintained somewhat higher PA levels over time (Figure S1), the direct association of intervention allocation with PA‐profiles was minor. However, the intervention seemed to encourage already physically active individuals to sustain their activity levels, albeit with limited impact on less active individuals. Prior evidence regarding interventions designed to increase total PA in older adults remains inconclusive [53]. While many systematic reviews and meta‐analyses have shown favorable effects of intervention on mean activity levels in short term [54, 55], the maintenance of PA beyond 1 year is unclear. Our study, utilizing person‐centered analyses, thus provides novel insights into PA trajectories among older adults in lifestyle intervention trials.

### 4.1 The Determinants of the PA‐Profiles

Surprisingly, the sociodemographic factors had only marginal associations with PA‐profiles. Previous research has shown that higher age and lower education predicts lower engagement in PA [5, 56], whereas in our study, education was not associated with the different PA‐profiles, and higher age only slightly increased the likelihood of belonging to *Moderate—sharply declining profile*. Moreover, in discord with prior studies [56, 57], we found that men were more likely than women to belong to *Constantly low profile*. On the other hand, they were less likely to be members of the *Moderate—declining profile*. The weak associations found between the sociodemographic factors and the PA‐profiles may partly be explained by sample homogeneity compared to the general population.

Similarly, while previous research shows that physically active older adults are more likely to preserve cognition than those who are physically inactive [13, 14, 15], we did not observe an association between cognition and the PA‐profiles. Prior studies have shown lower PA levels among people with cognitive decline [58, 59], but these studies were cross‐sectional, thus unable to conclude whether cognitive decline preceded or followed low PA.

Our analysis revealed that having depressive symptoms or pain increased the likelihood of membership in the *Constantly low profile* and decreased the likelihood of membership in the *High—stable profile*. Consistent with existing literature, the observed associations underscores their role as barriers of PA [7, 10, 57]. Although the associations were modest, they can be very significant at the population level due to the high prevalence of pain and depression in the older adults (Zimmer et al., 2020). Although, we investigated pain and depression as separate determinants of PA, it is likely that there are time‐varying causal chains between the investigated barriers, which future studies should address.

The intervention did not significantly modify the association between examined barriers and PA profiles, as indicated by the nonsignificant interaction terms (Table 2), although some effect modification was observed for PA motives.

### 4.2 PA Motives

To understand which PA motives make older adults initiate and sustain a physically active lifestyle, we examined various reasons for engaging in PA, their intercorrelation, and their association with longitudinal PA profiles. Our data‐driven approach identified three principal components encompassing different motives for PA (Table S1) that also predicted membership in distinct PA profiles.

*Distal and proximal benefits of exercise* included immediate and long‐term benefits. The short‐term, psychosocial advantages of PA (enjoyment, and improved social interaction, well‐being, and mood) reflect the immediate or proximal benefits, while positive health effects represent the long‐term, distal advantages. A high score on this component strongly predicted membership in the favorable PA profiles (Table 2); individuals that reported both distal and proximal benefits as main motives were more active and likely to maintain high activity levels over time. This aligns with findings from prior systematic literature reviews of qualitative studies, where social interaction, enjoyment, and recognition of physical health benefits were consistently noted as strong motivational influences for older adults to remain physically active [31, 34, 60].

Although our approach to analyzing PA motives was data driven, several theoretical frameworks suggest that the reasons individuals choose to engage in PA can be underpinned by underlying psychological and emotional drivers. For instance, the self‐determination theory (SDT) [61] posits that behaviors like PA are driven by motivational processes along a continuum from extrinsic to intrinsic regulation. Intrinsic motivation involves engaging in PA for the associated inherent pleasure (e.g., enjoyment), while extrinsic motivation stems from external pressures or rewards (e.g., exercising due to medical advice). Extrinsic motivation can be further divided into autonomous or controlled forms of motivation depending on the degree of internalization. According to SDT, long‐term engagement in PA is most likely when individuals experience autonomy (a sense of control over the activity), competence (confidence in their ability to perform and improve in PA), and relatedness (feeling connected to others through PA) [61].

In the current study, *Distal and proximal benefits of exercise* included motives aligned with intrinsic motivation (exercise in fun) or personal values and utility, reflecting a more autonomous form of behavioral regulation [62]. This supports the positive association between this component and the optimal PA profiles, consistent with SDT. The second component, *Appearance and media influence*, included appearance‐related reasons and media‐driven pressures to exercise, indicating more controlled forms of motivation. Although this component also correlated with favorable PA profiles, the associations were significantly weaker than those observed for the first component, aligning with SDT predictions. The third component *Disease management*, included health management reasons and professional guidance, reflecting more controlled than autonomous motives. This component showed varied associations with PA profiles, influenced by intervention participation. In the control group, those with high scores on *Disease Management* were more likely to belong to profiles where PA either remained stable or decreased substantially below average levels. This trend was not observed among those who participated in the intervention program. Participating in professionally guided group exercise may have enhanced feelings of autonomy, competence, and social connection, which may have led to long‐term changes in PA.

### 4.3 Strengths and Limitations

This study is based on a large, long‐term randomized controlled trial design with a high response rate, which is a key strength of this study. The design enabled us not only to examine the effect of the intervention on longitudinal PA profiles but also to investigate a range of barriers and incentives of PA and how the intervention modified their effect on PA engagement. The use of a person‐centered modeling strategy with repeated measurements of PA is another major strength as a conventional variable‐centered approach would not have captured the variability in the development of PA.

Limitations of this study include classifying the participants into the derived profiles based on posterior probabilities, which may have attenuated the actual effects [63]. Overall, the sufficiently high posterior probabilities and entropy (Table S1), however, indicated that the profiles were well separated [52], i.e., the groups clearly differed in terms of their PA trajectories. Any conclusions should, however, account for the fact that the profiles are not directly observed but are derived under certain modeling assumptions. Moreover, PA was assessed at five time‐points over the seven‐year follow‐up, so the data may not fully capture participants' PA during the intervals between the assessments, which is a limitation of our study. In future studies, more frequent follow‐up data are warranted.

Another point to consider is the generalizability of the results. The participants were recruited from population‐based cohorts, and they were similar to the general population in many characteristics [64]. However, healthy volunteer bias may limit the generalizability, as previous analyses have shown, that the invited non‐attendees were slightly older, less educated, less active, and had more chronic conditions than the participants [64]. The inclusion criteria on the other hand minimizes causality issues—none of the participants had disorders that could prevent participation in the intervention at baseline, and thus the factors that were investigated in this study can be considered to have preceded the development of disorders affecting PA. Furthermore, our attrition analyses (Table S3) revealed that people who dropped out during the follow‐up did not differ significantly from those included.

There are also certain limitations associated with the metrics used in the study. First, PA was based on self‐reports, which may entail recall bias. It is likely that this measure does not capture all the changes in PA (e.g., intensity and duration) introduced by the intervention program, aging of the participants or the examined barriers and motives. Moreover, the measure only accounted for PA lasting at least 20 min, whereas according to the current international PA guidelines (World Health Organization, 2020), the duration of each session of PA does not need to meet a specific minimum length to be health enhancing. Despite these limitations, studies have suggested that single‐item measures, such as that used in our study, are valid tools to determine the level of respondents' PA [65, 66] and can effectively capture changes in PA levels [67]. These measures provide a pragmatic balance between data accuracy and practicality, particularly in studies tracking changes over time rather than aiming at a precise quantification of PA. Future studies should, however, consider objective measures of PA, using wearable devices, to capture nuanced changes in PA. Second, while the PCA successfully derived distinct components with predictive validity for PA engagement, the questionnaire on PA motives was not externally validated. Because the motives PA were asked using predefined options, not all possible motives may have been captured. Additionally, the data‐driven approach lacking a theoretical underpinning to inform PA motives may be considered a limitation. Consequently, these results should be viewed as exploratory. Prior studies using validated questionnaires based, for example, on SDT theory may offer a more thorough understanding of motivations for engaging in PA.

### 4.4 Perspective

Encouraging older adults to engage and maintain PA in long‐term is a central public health focus. Maintaining PA is particularly important for older adults who have increased risk of dementia. This study is one of the few to longitudinally map PA profiles over an extended period, in an elderly population at‐risk of dementia, who participated in multidomain lifestyle intervention. The PA profiles shed light on the longitudinal patterns of PA among older adults, while their identified determinants provide actionable insights for public health professionals for tailoring interventions to maintain or increase PA, particularly in those most at risk of decline. The modest impact of the intervention on PA profiles highlights the complexity of behavioral change. The observed associations between depressive symptoms, pain, and PA profiles underscore the importance of addressing mental health and pain management in interventions aimed at increasing PA in older adults. The results also reinforce the understanding that social aspects, enjoyments, and health benefits are extremely important motives for maintaining PA levels in older adults.

## Author Contributions

K.J., N.T., and K.S. conceptualized the current study. K.S. conducted data analyses and wrote the original draft of the manuscript. N.T., L.T., S.T., A.R., T.J., S.H., and K.M. designed; and N.T. and L.J. coordinated the trial. K.M. is the principal investigator of the FINGER study. All authors reviewed and revised the article for intellectual content and approved the final manuscript.

## Conflicts of Interest

The authors declare no conflicts of interest.

## Supporting information


Appendix S1.

## Data Availability

The data presented in this article are not readily available because public deposition of the de‐identified dataset is not possible due to legal and ethical reasons, and complete deidentification is not possible as this investigation is part of an ongoing study. The study participants gave informed consent which includes data use only under confidentiality agreement. Further, the data contain large amount of sensitive information and public data deposition may pose privacy concerns. Those fulfilling the requirements for viewing confidential data as required by the Finnish law and the Finnish Institute for Health and Welfare are able to access the data after completion of material transfer agreement. Requests to access the datasets should be directed to kirjaamo@thl.fi.
