# Supplementary material for: Cognition, Depression, Pain, and Exercise Motives as Predictors of Longitudinal Profiles of Physical Activity During a Seven‐Year Follow‐Up Among Older Adults
Source: Scand J Med Sci Sports. 2024 Dec 13;34(12):e14777. doi: 10.1111/sms.14777 (PMC11638919; doi:10.1111/sms.14777)
Supplement: Supplementary file 1 — Appendix S1. [file SMS-34-e14777-s001.docx]

**PRINCIPAL COMPONENT ANALYSIS: MOTIVES FOR PHYSICAL ACTIVITY AMONG FINGER PARTICIPANTS**

To examine the physical activity (PA) motives, the participants were asked a question: “Which of the following factors encourage you to exercise?” Respondents were instructed to select all those that applied to them from among twelve answer options (1 = positive health effects, 2 = encouragement from healthcare professionals, 3 = disease management, 4 = improvement of well-being and mood, 5 = family and loved ones exercise, 6 = positive effects on appearance, 7 = information about exercising obtained from the media, 8 = meeting friends, 9 = exercising is trendy, 10 = exercising is fun, 11 = other reason, specify, 12 = nothing). For the analyses option 11 was excluded.

A tetrachoric correlation matrix was created for the dichotomous items describing the motives, which showed correlation between the items. Principal component analysis (PCA) was used to reduce the items into fewer components. Varimax rotation was employed for the PCA to obtain the best fitting solutions, and factor coefficients were used to obtain the factor scores for the extracted components. The number of the extracted components was based on interpretation of a scree plot, which illustrates the eigenvalues against component numbers, and eigenvalues >1. Component loadings higher than 0.4 were considered significant. Considering statistically significant value of Bartlett’s test of sphericity (107.56; p < 0.001), the data was deemed suitable for PCA (Sharma, 1996). The Kaiser–Meyer–Olkin (KMO=0.73) measure of sampling adequacy showed that the measures used in the study are valid for analyzing PA motives.

**Results**

PCA showed three patterns of PA motives in the whole sample, which explained 66.1% of the total variance in all the items. Component 1 had heavy positive loadings on ‘positive health effects’, ‘improves well-being and mood’, ‘exercising is fun’, ‘family and loved ones exercise’, and ‘meeting friends’, on the other hand, ‘nothing motivates me to exercise’ had a strong negative loading (i.e., participants selecting the above mentioned motives were the least likely to report that nothing motivated them to engage in physical activity). This component was named *Distal and proximal benefits of exercise*. The second component, in turn, loaded heavily on external or appearance-driven motives influenced by societal trends and media (‘exercising is trendy’, ‘positive effects on appearance’, and ‘information about exercising obtained from the media’), and was named accordingly *Appearance and media influence.* The third component had heavy loadings on ‘disease management’ and ‘encouragement from healthcare professionals’ and was named as *Disease management.*

**Supplementary Table 1.** Principal component analysis of items describing motives for physical activity among participants of the Finnish Geriatric Intervention Study to Prevent Cognitive Impairment and Disability (FINGER)

| **Motivation factor:** | **Component 1:**  **Distal and proximal benefits of exercise** | **Component 2:**  **Appearance and media influence** | **Component 3:**  **Disease management** |
| --- | --- | --- | --- |
| Nothing encourages me to exercise | -0.93 |  |  |
| Positive health effects | 0.76 |  |  |
| Improves well-being and mood | 0.74 |  |  |
| Exercising is fun | 0.59 |  |  |
| Family and loved ones exercise | 0.54 |  |  |
| Meeting friends | 0.51 |  |  |
| Exercising is trendy |  | 0.88 |  |
| Positive effects on appearance |  | 0.74 |  |
| Information about exercising obtained from the media |  | 0.68 |  |
| Disease management |  |  | 0.82 |
| Encouragement from healthcare professionals |  |  | 0.79 |
| Variance explained, % | 28.5 | 23.0 | 14.6 |

*Note.* Extraction method principal component analysis employing varimax rotation. Bartlett’s test p-value <0.001. Total variance explained by the extracted components = 66.1%

**General estimation equation: The overall trajectories of physical activity in the intervention and control groups**


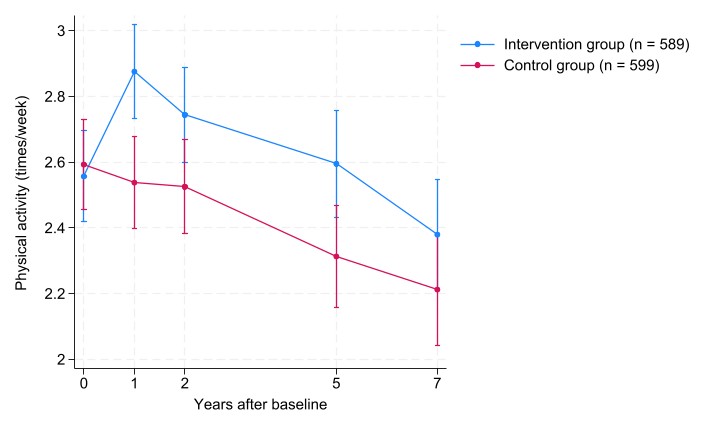


**Figure S1** Estimated frequency of physical activity with 95% CIs during a seven-year follow-up among participants of the Finnish Geriatric Intervention Study to Prevent Cognitive Impairment and Disability (FINGER)

**Detailed description of the conducted latent class growth curve analysis (LCGA)**

Latent class growth analysis was used to identify latent groups of participants with similar PA trajectories over the seven-year follow-up. LCGA is a finite mixture model that uses maximum likelihood estimation to identify groups of individuals that follow similar development of a trait over time (Muthen, 2004). In LCGA the within-group variation is fixed to zero to aid in identification of the classes. (Berlin et al. 2014).

The best fitting model was searched through an iterative analytic process (Nagin, 2005; Nylund et al., 2007). First, models with increasing number of latent classes with a quadratic-shaped trajectories were fitted until statistical fit indices showed no improvement in model fit. Models with 1 to 7 classes were tested. Next, whether the fit of the chosen model improved with adding or reducing the polynomial order of each trajectory was tested. After inspecting the derived trajectories, we chose the most parsimonious model, that showed subjectively meaningful trajectories. I.e., the best fitting model was based on the subjective interpretability of the trajectories, size of the classes (>5%) and statistical fit indices, including the Bayesian information criterion (BIC) (lowest value), AIC (lowest value), posterior probabilities of class membership >0.80, and the entropy value >0.70 (Berlin et al. 2014). For each subject, the model provides the probability of belonging to each of the identified trajectory classes and assigns them to the class based on the highest probability. The class membership was then used as the independent variable at the next step of the analysis. The fit indices are shown in Supplementary Table 2, and the estimated class proportions and parameter estimates from the chosen six class model are presented in Supplementary Table 3.

**Supplementary Table 2.** Model fit indices of latent class growth analysis: Physical activity profiles among the participants of the Finnish Multidomain Intervention Study to Prevent Cognitive Decline and Disability (FINGER) during a seven-year follow-up (n = 1188)

| Number of trajectories | Polynomial order | BIC | AIC | Entropy | Average posterior probabilities | Group size |
| --- | --- | --- | --- | --- | --- | --- |
|  |  |  |  |  |  |  |
| 1 | 2 | -9645.05 | -9634.89 | - | 1 | 1 |
| 2 | 2 2 | -9073.62 | -9053.29 | 0.75 | 0.90/0.94 | 0.34/0.66 |
| 3 | 2 2 2 | -8813.12 | -8782.64 | 0.81 | 0.91/0.92/0.88 | 0.19/0.63/0.18 |
| 4 | 2 2 2 2 | -8772.06 | -8731.42 | 0.71 | 0.89/0.80/0.80/0.87 | 0.15/0.37/0.39/0.10 |
| 5 | 2 2 2 2 2 | -8756.53 | -8705.73 | 0.73 | 0.89/0.79/0.73/0.80/0.88 | 0.15/0.40/0.04/0.33/0.08 |
| 6 | 2 2 2 2 2 2 | -8738.17 | -8677.21 | 0.73 | 0.83/0.74/0.82/0.73/0.80/0.83 | 0.11/0.11/0.49/0.05/0.18/0.06 |
| 7 | 2 2 2 2 2 2 2 | -8734.63 | -8663.51 | 0.69 | 0.81/0.79/0.69/0.69/0.77/0.78/0.79 | 0.13/0.03/0.17/0.04/0.45/0.13/0.06 |
| **6** | **2 1 2 2 0 0** | **-8722.77** | **-8674.51** | **0.71** | **0.82/0.72/0.80/0.73/0.77/0.88** | **0.12/0.09/0.47/0.05/0.22/0.06** |

*Notes.* AIC= Akaike Information Criterion, BIC= Bayesian Information Criterion

**Supplementary Table 3**. Estimated class proportions and parameter estimates from the chosen six class model of latent class growth analysis: Physical activity profiles among the participants of the Finnish Multidomain Intervention Study to Prevent Cognitive Decline and Disability (FINGER) during a seven-year follow-up (n = 1188)

|  | **N** | **% of total** | **Intercept, mean (S.E.)** | **Linear term, mean (S.E.)** | **Quadratic term, mean (S.E.)** |
| --- | --- | --- | --- | --- | --- |
| **Profile 1** | 137 | 12 | -1.194 (0.276) | 0.739 (0.418) | -0.319 (0.157) |
| **Profile 2** | 104 | 9 | 0.190 (0.412) | 0.131 (0.084) | NA |
| **Profile 3** | 564 | 47 | 2.593 (0.190) | 0.099 (0.066) | -0.027 (0.009) |
| **Profile 4** | 56 | 5 | 2.670 (0.396) | 1.094 (0.765) | -0.642 (0.356) |
| **Profile 5** | 257 | 22 | 4.225 (0.336) | NA | NA |
| **Profile 6** | 70 | 6 | 7.193 (0.563) | NA | NA |

*Notes.* S.E. = standard error. NA = not applicable (the polynomial term was omitted for the profile in question)

**ATTRITION ANALYSIS**

**Supplementary Table 3.** Baseline differences between included and excluded participants.

|  | Included ^a^  (n = 1188) | Lost to follow-up ^b^  (n = 71) | p for difference |
| --- | --- | --- | --- |
| Age, years, mean (SD) | 68.8 (4.7) | 69.3 (4.9) | 0.421 |
| Women, % | 46 | 54 | 0.230 |
| Education, years (SD) | 10.0 (3.4) | 9.5 (3.8) | 0.236 |
| Intervention group, % | 50 | 59 | 0.117 |
| Cognition (NTB z-score) (SD) | -0.00 (0.57) | -0.05 (0.61) | 0.476 |
| Depressive symptoms (Zung total score) (SD) | 0.00 (1.00) | 0.00 (1.00) | 0.985 |
| Pain (z-score) (SD) | -0.00 (0.99) | 0.07 (1.10) | 0.531 |
| Physical activity Motives |  |  |  |
| Distal and proximal benefits of exercise (z-score) (SD) | 0.56 (0.27) | 0.48 (0.31) | 0.026 |
| Appearance and media influence (z-score) (SD) | 0.26 (0.30) | 0.24 (0.32) | 0.658 |
| Disease management (z-score) (SD) | 0.13 (0.38) | 0.16 (0.38) | 0.534 |

*Notes:* Summary statistics calculated among participants with non-missing data. ^a^ missing data on education (n = 1), cognition (n = 1), depressive symptoms (n = 124), pain (n = 10); ^b^ missing data on education, (n = 1), depressive symptoms (n = 10), pain (n = 1)

**REFERENCES:**

Sharma (1996) Sharma S. Applied multivariate techniques. John Wiley & Sons, Inc; New York: 1996.

Muthén BO (2004) Latent variable analysis: growth mixture modeling and related techniques for longitudinal data. In: Kaplan D (ed) Handbook of quantitative methodology for the social sciences. Sage Publications, Newbury Park

Berlin KS, Parra GR, Williams NA. An introduction to latent variable mixture modeling (part 2): longitudinal latent class growth analysis and growth mixture models. J Pediatr Psychol 2014;39:188–203. doi:10.1093/jpepsy/jst085.

Nagin, Daniel S.. Group-Based Modeling of Development, Cambridge, MA and London, England: Harvard University Press, 2005. https://doi.org/10.4159/9780674041318

Nylund KL, Asparouhov T, Bengt MO (2007) Deciding on the number of classes in latent class analysis and growth mixture modeling: a Monte Carlo simulation study. Struct Equ Model 14(4):535–569. https://doi.org/10.1080/10705510701575396
